# Perspective use of bio-adhesive liquid crystals as ophthalmic drug delivery systems

**DOI:** 10.1038/s41598-023-42185-z

**Published:** 2023-09-27

**Authors:** Martine Tarsitano, Antonia Mancuso, Maria Chiara Cristiano, Konrad Urbanek, Daniele Torella, Donatella Paolino, Massimo Fresta

**Affiliations:** 1 Department of Health Science, University Magna Graecia of Catanzaro, Campus Universitario-Germaneto, Viale Europa, 88100 Catanzaro, Italy; 2 Department of Experimental and Clinical Medicine, University Magna Graecia of Catanzaro, Campus Universitario-Germaneto, Viale Europa, 88100 Catanzaro, Italy; 3 Department of Medical and Surgical Sciences, University Magna Graecia of Catanzaro, Campus Universitario-Germaneto, Viale Europa, 88100 Catanzaro, Italy; 4 Department of Molecular Medicine and Medical Biotechnologies, University of Naples “Federico II”, Via A. Panzini 5, 80131 Naples, Italy; 5 CEINGE-Advanced Biotechnologies, Via G. Salvatore 486, 80131 Naples, Italy

**Keywords:** Drug delivery, Pharmaceutics

## Abstract

The success of many drugs in ophthalmic treatments is hindered by their physico-chemical properties and the limited precorneal retention time. Here, lyotropic liquid crystals are proposed as a new ophthalmic drug delivery system. Acyclovir was chosen as model drug for its solubility and its controlled release from cubic phase was achieved. We demonstrated the effortless application of lamellar phase on corneal surface and its ability to convert itself in cubic phase in situ. While the complex viscosity of lamellar phase was affected by temperature (5.1 ± 1.4 kPa·s at 25 °C and 0.12 ± 0.001 Pa·s at 35 °C, respectively), the cubic phase shown no changes in viscosity values and shear thinning behaviour at both temperatures and even in presence of the drug The degradation kinetic of drug-loaded cubic phase was slightly slower than the empty formulation, recording 27.92 ± 1.43% and 33.30 ± 3.11% of weight loss after 8 h. Ex vivo studies conducted on porcine eyeballs and isolated cornea confirmed the instantaneous transition to cubic phase, its ability to resist to gravity force, and forced dripping of simulated tear fluid. Histopathological investigation showed how treated cornea did not report changes in epithelial and stroma structures. In summary, lyotropic liquid crystals could represent an advantageous ophthalmic drug delivery system.

## Introduction

Topical administration of eye drops is the most common way to treat ocular diseases due to the presence of blood–retina barrier that renders ineffective the systemic administration of drugs. Unfortunately, the drugs that can be effectively administered in conventional ocular dosage forms are very few. The reduced availability of effective ophthalmic treatments is due certainly to the physico-chemical features of drug(s) such as solubility, logP, bioavailability, shelf life, but also due to the physio-anatomical characteristics of eyes. The main limit could be considered the ocular physiological protective apparatus, represented by reflex ciliary movement and constant lachrymal flux, whose physiological task is to eliminate foreign components from the eye^1^. This rapid elimination, typically for ophthalmic conventional solution or suspension, resulted in the need for repeated administrations and high doses of drug; as well as systemic side effects due to fast nasolacrimal drainage^2^. In this scenario, a bioadhesive matrix system could be able to prolong the interaction time between drug(s) and ocular structures, inhibiting the rapid pre-corneal elimination. Several studies were carried out to evaluate the suitability of stimuli responsive hydrogels as ophthalmic drug delivery systems. For example, hydrogels responding to temperature^3^, pH^4^, ionic environment^5^ were proposed as in situ gelling hydrogels, because their precursor solutions were able to induce a rapid in situ formation of 3D-matrix. It must be considered that these stimuli are not always controllable and predictable, above all when patients are subjected to other treatments such as laser, or lens uses, or in presence of some diseases that can modify ocular physiological features^6^. In some cases, the combination of pH and temperature-responsive polymers can optimise the ophthalmic dosage form^7^, greatly complicating the composition of the formulation.

Starting from the need to extend the contact time of the formulation on ocular surface and to increase the ocular permeation of drug, we are proposing lyotropic liquid crystals as instantaneous in situ forming cubic phase for ophthalmic application. When some polar amphiphilic lipids, such as glyceryl monooleate, are placed in contact with aqueous medium (water, phosphate buffer solution, simulate tear fluid, etc.), they can re-organize themselves into different structures, starting from bilayers typically for lamellar phase until a more organized internal structures characterizing cubic phase^8^.

Lyotropic liquid crystals are versatile delivery systems whose composition can be modified, simplified, or enriched according to specific purpose. Lyotropic liquid crystals are mesophase with intermediate properties between liquids and solids^9^. Generally, they are described as a mixture of amphiphilic compounds able to modify their internal structure and their properties as a function of a single and specific stimulus which is the added amount (%w/w) of solvents. The main advantage of using the cubic phase as drug delivery systems is its great stability and the possibility to predict the drug release. In detail, the cubic phase, obtained by hydrating the precursor lamellar phase with an excess aqueous medium, is able to resist at a wide range of pH or temperature variations without modifying its structure and its properties^10,11^. Its structure is characterised by bicontinuous lipid bilayers separated by water channels^12^; this amphiphilic nature permits to deliver drugs with different physico-chemical features and the release mechanism is strongly dependent on drug properties, because a hydrophilic drug could tend to rapidly diffuse within water channel, while a lipophilic drug could tend to strongly interact with lipid constituents of cubic phase and its release resulted slower and depending by matrix degradation. Cubic phases are described by several research groups as thermodynamically stable, optically isotropic, highly viscous, and transparent^13^, and the researchers are proposing this matrix above all for skin drug delivery; unfortunately, the promising properties of the cubic phase are not sufficient to allow their use for all administration routes, considering that cubic phase administration may not be easy. A solution could be the use of precursor lamellar phase that become gel-like when exposed to excess hydration medium. In fact, the lamellar phase is characterised by planar lipid bilayers arranged in a one-dimensional structure. It appears less viscous, less rigid, and easier to administer, but also more sensitive to stimuli, such as temperature variation, component addiction, etc., in comparison with cubic phase.

The idea to use lyotropic liquid crystals and to propose them for ophthalmic application was born from the main advantages of lamellar and cubic phases: the easy administration of the lamellar phase, its instant transformation into cubic when placed in contact with the excess of water and the high stability and controlled release of the cubic phase. To study the features of lyotropic liquid crystals and to better understand their possible application in ophthalmic approach, we prepared lamellar phase and consequently cubic phase containing acyclovir, chosen as model drug. Acyclovir is an antiviral drug active against herpes simplex virus infection; it is used for ophthalmic infection treatments, but it is prescribed in form of ointment and not as drops because of its low water solubility and poor bioavailability^14^. Moreover, the effectiveness of conventional acyclovir ointments, solution or suspension is limited by blinking and tears that tend to remove the formulation^15^. Consequently, to reach a suitable ocular drug absorption is very difficult by using these conventional dosage forms. On the contrary, the ophthalmic application of acyclovir-loaded lamellar phase that becomes a cubic structure in contact with tear fluid could be an effective approach for the treatment of several ocular diseases. To confirm this concept, in this study we performed a deep rheological characterization of lamellar and cubic phase, we evaluated the acyclovir release profile and the degradation profile of cubic phase in presence of simulated tear fluid and, finally, we carried out ex-vivo studies on porcine cornea to confirm the safety ocular profile of formulation comparing it with a chemically-induced damage on isolated corneal tissue^16^.

## Results and discussion

### Physico-chemical and rheological characterization of empty and acyclovir-loaded lyotropic liquid crystals

**Figure 1:**
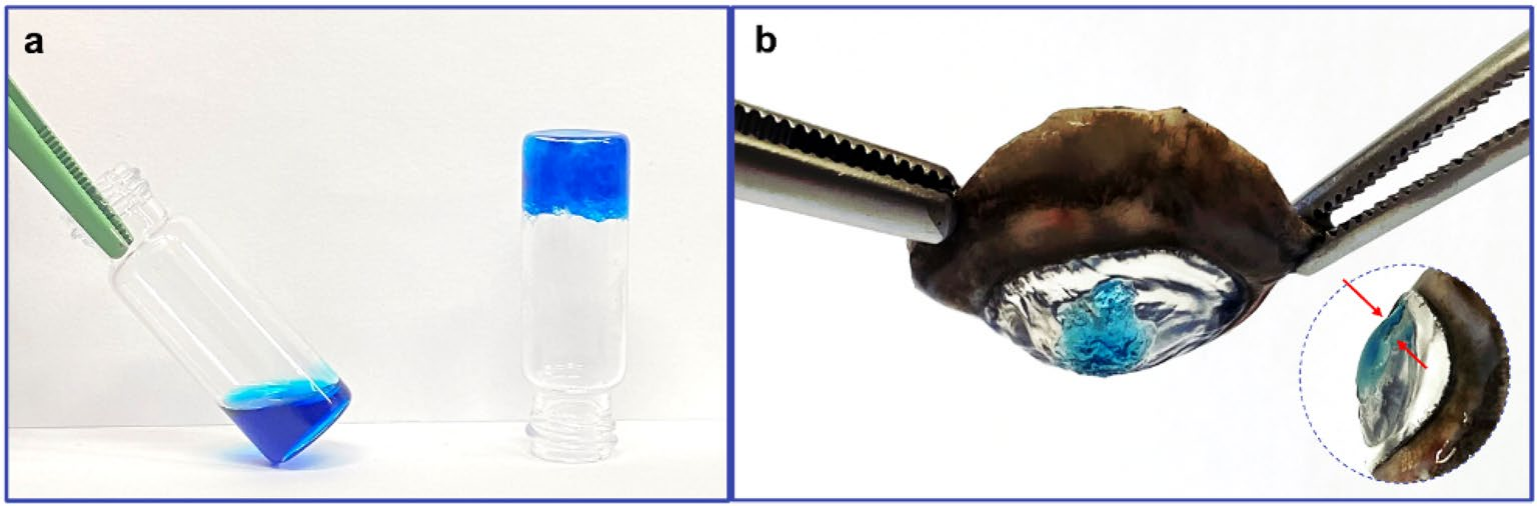
Illustrative photography of lamellar (on the left of panel (**a**)) and cubic phase (on the right of panel (**b**)) prepared by using methylene blue as a probe for better visualization of the samples. Panel (**b**) shows the in situ formed cubic phase on isolated porcine cornea, and the red arrows indicate the thickness of the cubic phase.

The effectiveness of traditional ophthalmic therapy is limited by the rapid elimination of the eye drops, resulting in a short duration of the therapeutic effect of instilled drugs. In addition, the physiological tear drainage induces the passage of a part of the administered drug via nasolacrimal duct into the gastrointestinal tract, often inducing side effects^17^. The in-situ administration of drug is the main route for the treatment of local ocular diseases, and considering the limitation of traditional approaches, we proposed in this research work the innovative use of lyotropic liquid crystals for ophthalmic delivery of acyclovir. As matrix systems, lyotropic liquid crystals are able to modify their physical and morphological structure, passing from lamellar to cubic phase, as response to an increase in solvent availability. Lamellar phases were obtained by adding a specific amount of water to the pre-heated amphiphilic glyceryl monooleate (GMO), by using a very easy preparation method. The obtained samples were analysed, evaluating their rheological behaviour, and subsequently they were transformed in cubic phases, placing the precursor solutions in contact with excess medium, thus obtaining a gel-like structure (Fig. 1a). In detail, the simulated tear fluid was used as aqueous medium, mimicking what would happen if the lamellar phases were administered into the human eye. The liquid lamellar phase was applied on isolated porcine cornea and its transition to cubic phase was instantaneous and easily visible to the naked eye (Fig. 1b). Rheological profiles confirmed significant differences in terms of complex viscosity and temperature-dependence between the two phases.

**Figure 2:**
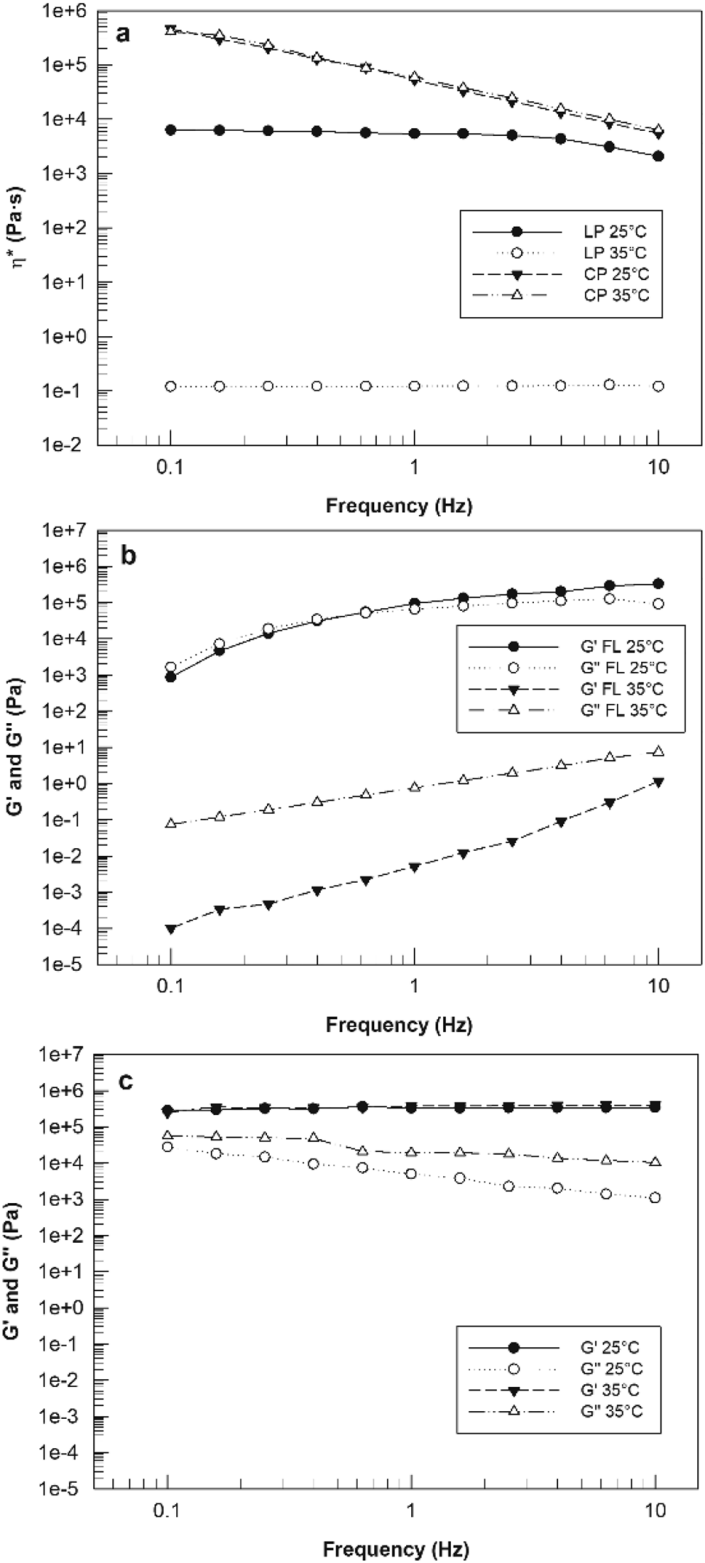
Rheological characterization of lyotropic liquid crystal obtained by using Kinexus Rotational Rheometer. Panel (**a**) shows complex viscosity (η*—Pa·s) curves of lamellar and cubic phase of GMO-based lyotropic liquid crystal. Dynamic moduli change of lamellar (panel (**b**)) and cubic phase (panel (**c**)) of lyotropic liquid crystals was represented as a function of temperature and frequency. The data were expressed as a function of temperature and frequency. The result was representative of five independent experiments.

Rheological studies are considered a suitable analysis for characterizing lamellar and cubic phase^18^. Figure 2a reports the curve of complex viscosity as a function of frequency for lamellar and cubic phases, analysed at 25 °C and 35 °C. The different rheological response of two samples to temperature changes is immediately evident. Complex viscosity profile of lamellar phase showed a great dependence on temperature; in fact, as can be seen in Fig. 2a (circle symbols), the complex viscosity profile of lamellar phase at 25 °C was much higher than the curve obtained from the sample analyzed at ophthalmic temperature. The average values of the complex viscosity were 5.1 ± 1.4 kPa·s and 0.12 ± 0.001 Pa·s, respectively for lamellar phase samples analyzed at 25 °C and 35 °C.

Contrary to lamellar phase, the complex viscosity profile of the cubic phase (triangle symbol, in Fig. 2a) was not influenced by temperature change: the curves of complex viscosity vs frequency obtained at 25 °C and 35 °C were overlapped. This result confirmed the thermodynamic stability of the cubic phase, which appeared as a highly viscous sample^19^ (Fig. 1a). Moreover, a shear-thinning behaviour was shown by the cubic phase sample, i.e., complex viscosity values varied as a function of applied solicitation^13^ (Fig. 2a). This shear-thinning behaviour was not shown in lamellar phase profile; in this case, the samples showed an almost Newtonian profile: at low frequency values, the complex viscosity values of lamellar phase, regardless of the used temperature, seemed to be not altered from applied solicitation^20^. Another important difference can be noted comparing viscosity values of lamellar and cubic phases. Above all at 35 °C, the complex viscosity values related to cubic phase were significantly higher than values of lamellar phase; this increased viscosity in cubic phase sample was probably due to the increased organization of liquid crystal microstructure, that was characterized by a three-dimensional order when cubic phase was formed^21^. Instead, lamellar phases with one-dimensional long-range order were characterized by a fairly low viscosity.

The different response of rheological behaviour of samples to temperature changes was evident also in Fig. 2b,c, reporting G′ and G″ curves as a function of frequency and temperature. It is important to note that, when temperature was increased from 25 °C until ophthalmic temperature, the loss modulus G″ of lamellar phase clearly dominated the storage modulus G′ (Fig. 2b); this ratio between G′ and G″ indicates a predominant “liquid-like” behaviour^22^ and confirms the reduction of viscosity recorded when lamellar phase was analysed at 35 °C (Fig. 2a). On the contrary, the curves of G′ and G″ moduli, related to lamellar phase analysed at room temperature, followed the same trend appearing very close to each other. Also this result confirmed the sensitivity of lamellar phase at the analysis temperature, clearly distinguishing it with the cubic phase obtained by hydration. The Fig. 2c reports the storage modulus G′ and the loss modulus G″ curves of cubic phase; we can note that G′ modulus curves remained above the G″ curves for the whole used frequency range and at both temperatures of analysis, showing a predominant “solid-like” behaviour^23^. Moreover, the figure shows as the G′ curve related to cubic phase maintained at 25 °C was perfectly overlapped on the G′ curve obtained maintaining the cubic phase at 35 °C, confirming once again the resistance of cubic phase when temperature was modified.

Considering that we proposed an in situ forming cubic phase for ophthalmic drug release, it was important to evaluate the possibility to obtain sterile samples. For this reason, after their preparation, lamellar phases were subjected to an autoclaving process and rheological analysis was used as reference evaluating any change in complex viscosity.

The data reported in Supplementary Table S1 showed that the autoclaving process induced significant variation in complex viscosity of lamellar phase. Despite this we can confirm the possibility to undergo the lamellar phase to autoclaving process, because the slight reduction in complex viscosity observed after the sterilization of the lamellar phase can be considered advantageous, making administration even easier. Moreover, the autoclaving process did not alter the ability of the lamellar phase to convert itself in the cubic phase when exposed to excess medium.

Lyotropic liquid crystals are considered suitable drug delivery systems for several applications, but their structure is greatly influenced by the phase composition, as the presence of additives, stabilizers, solvents etc.^24,25^. A drug, as acyclovir, could be considered as an additive and it could modify physico-chemical properties of lamellar and cubic phase, in terms of water uptake, degradation profile and rheological behaviour. For this reason, the proposed ophthalmic lyotropic liquid crystals in the form of lamellar and cubic phase were characterized also after loading of acyclovir, which was chosen as a model of drugs for ophthalmic use.

The obtained results confirmed that the transition from lamellar phase to cubic one in presence of simulated tear fluid occurred and it was instantaneous both in empty and acyclovir-loaded lyotropic liquid crystals. However, the amount of fluid necessary to induce the formation of the cubic phase was influenced by the presence of acyclovir. The evaluation of TFU (tear fluid uptake) highlighted that the drug solubilized within the lamellar phase induced the formation of the cubic phase by using a reduced amount of simulated tear fluid (74.42 ± 1.39%) compared to empty lamellar phase (85.55 ± 1.83%). Acyclovir is characterized by low lipophilicity with a log P equal to − 1.76, but it is also slightly soluble in water (1.4 mg/mL at room condition)^26^. Probably, these properties induced a particular disposition of drug within the water channel and lipid layers of lamellar and cubic phase occupying some of the slots normally engaged in the bonds between water and GMO. The possibility to obtain acyclovir-loaded cubic phase by using less amount of simulated tear fluid permitted to hypothesize its easier and guaranteed in situ formation.

Despite these differences in terms of water uptake, the rheological profiles of acyclovir-loaded phases showed a similar trend to the profiles of empty samples, both for lamellar and cubic phase and regardless of the temperature used for analysis.

**Figure 3:**
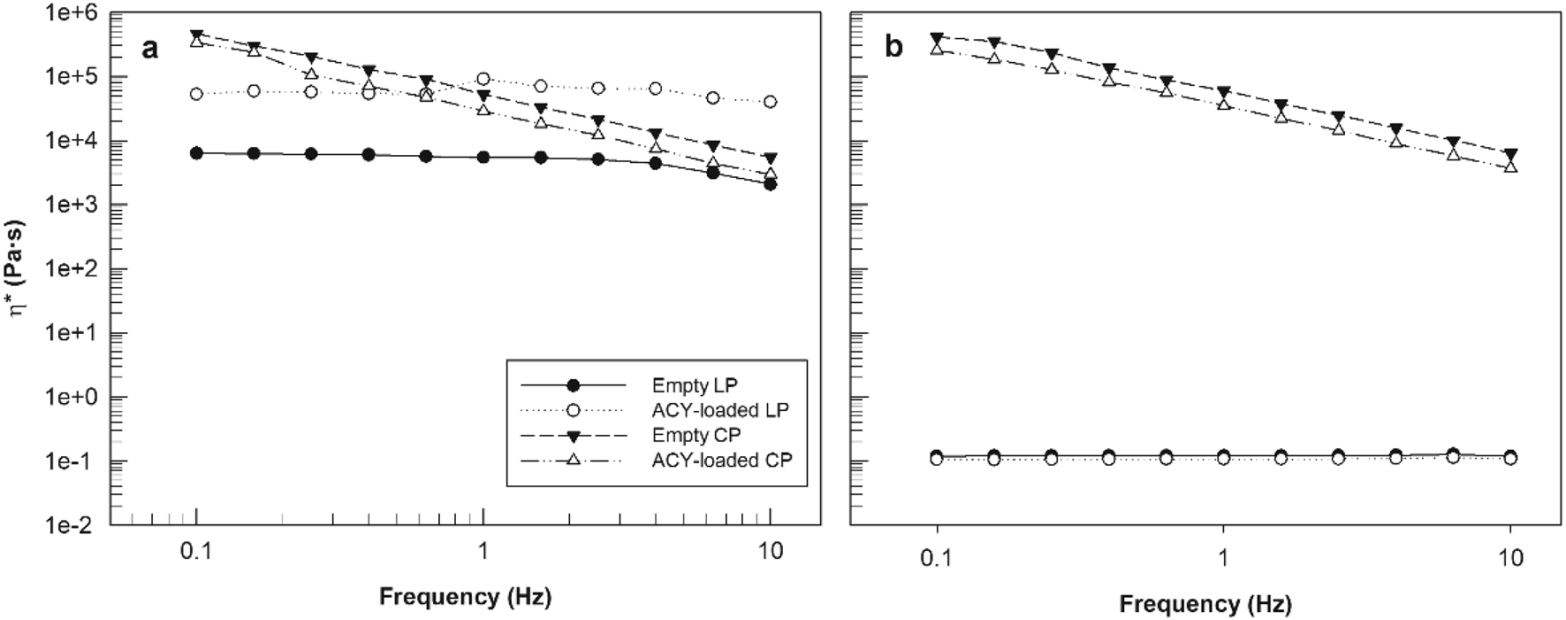
Complex viscosity (η*—Pa·s) curves of empty and Acyclovir (1 mg/g) loaded-lamellar and cubic phases of GMO-based lyotropic liquid crystal, obtained at 25 °C (**a**) and 35 °C (**b**). The data were expressed as a function of frequency. The result was representative of five independent experiments.

Figure 3 shows that the cubic phase prepared with Acyclovir (1 mg/g) responded to solicitation and temperature changes exactly as empty cubic phases, confirming our previous results obtained using probes that mimic the behavior of drugs with different solubility properties^27^. The complex viscosity profiles of acyclovir-loaded cubic phase were not influenced by temperature, and they are almost overlapped on complex viscosity profiles of empty cubic phase, at 25 °C and 35 °C. About lamellar phases, also in presence of acyclovir, their complex viscosity values were strongly influenced by temperature change, confirming the data obtained for empty lamellar phase. In detail, the average complex viscosity values for acyclovir-loaded lamellar phases were 59 ± 13 kPa·s and 0.11 ± 0.001 Pa·s, respectively recorded at 25 °C and 35 °C.

Comparing the complex viscosity profiles of empty lamellar phase and acyclovir-loaded lamellar phase, we can note that the viscosity of the latter is greater than the viscosity of the empty sample (Fig. 3a). We hypothesized that this effect was due to the amount of acyclovir added during the preparation phase; the percentage of acyclovir used in this study was the maximum soluble amount within water and GMO solution, and it saturated the formulation. Probably, this drug acted as additive and in particular as a thickener, able to induce an increase in viscosity of lamellar phase. Even if this effect was already visible to the naked eye and confirmed by rheological study at room temperature, it did not affect the viscosity results of lamellar phases carried out at the ocular temperature, thus confirming that the presence of the drug did not impact the temperature influence on samples.

**Figure 4:**
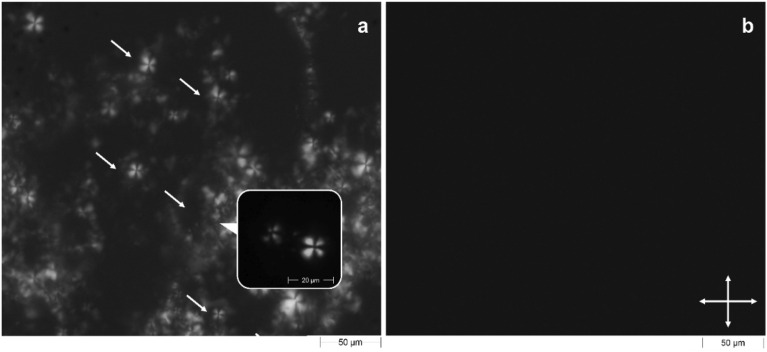
Morphological characterization of acyclovir-loaded lamellar and cubic phases. Microscopies of lamellar (**a**) and cubic (**b**) phases, containing acyclovir, were obtained using the Morphologi G3S equipped with polarizing filters. Observations were performed using a magnification 10 × or 50 ×. Crossed white double-arrows indicate the orientation of polarizers.

For further characterizing acyclovir-loaded lamellar phase, polarized light microscopy has been used^28^. This technique is normally useful to identify the optical isotropy or anisotropy of lyotropic liquid crystals^29^. Each liquid crystals show typical black and white texture when they are able to show birefringence such as in lamellar phase form. As we can see in Fig. 4a, the acyclovir-loaded lamellar phase showed a dominant texture characterized by Maltese crosses, also known as cruciate flowers, which are typical for this type of phase^30^. The absence of flowers or any white texture on dark background in Fig. 4b confirmed the presence of a cubic phase, because it is an isotropic phase whose internal structure did not show birefringence when it was hit by polarized light.

Since acyclovir affected the complex viscosity profile of lamellar phases without preventing the formation of cubic phase, we also evaluated its effect on time-dependent stability of samples. For this purpose, Turbiscan Lab^®^ Expert was used to evaluate during all time analysis (3 h) the Δ-backscattering and Δ-transmission profiles of empty and acyclovir-loaded lamellar phases when they were hit by a specific laser beam. The macroscopic evaluation, carried out monitoring samples integrity at naked eye, permitted to observe the persistence of a homogeneous appearance of the formulations during their conservation. However, the use of an instrument such as the Turbiscan Lab^®^ Expert allowed us to confirm the stability of the samples by a more accurate method.

In detail, the empty lamellar phase showed a good stability profile in terms of Δ-Backscattering (ΔBS) and Δ-transmission (ΔT), maintaining its curve within a suitable and stable range of ± 5 (Supplementary Fig. S1)^25^. The addition of acyclovir during lamellar phase preparation, unexpectedly, induced a further flattening of the curves on the baseline, highlighting a greater stability of the drug-loaded sample over time. We supposed that the partial solubility of acyclovir within GMO and its water solubility induced the formation of binds within the linear arrangement of lipid bilayers of lamellar phase^12^, permitting to strengthen the inner structure and reduce slippage between compartments.

The improved stability by adding acyclovir within lamellar phase can be considered an advantage: if we wish to consider future commercialization of acyclovir-loaded lamellar phase, it is important to obtain a product which is very stable during the storage period under standard conditions and which is able of transforming into an even more stable cubic phase when administered. The results obtained by rheological analysis and stability tests confirmed this point.

Another important feature to be evaluated was the degradation rate of cubic phase, in terms of weight loss (%) as a function of time. The degradation features of the system represent a fundamental parameter to be evaluated in relation to the intended purpose. In fact, if the formulation persisted in the ocular site for a time longer than that necessary to release the amount of delivered drug, this would represent an obstacle, reducing the patient compliance. We evaluated the weight loss profile of empty and Acyclovir-loaded cubic phases maintained in stirring and in contact with excess of simulated tear fluid.

**Figure 5:**
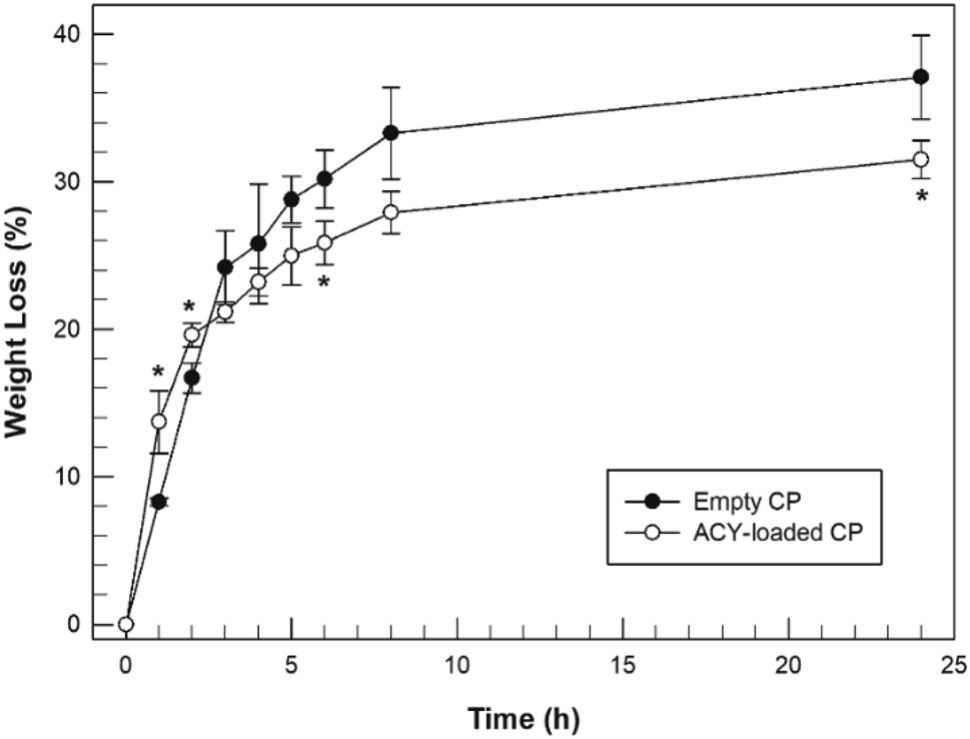
Degradation profile of empty (Empty CP—black circle) and Acyclovir-loaded cubic phases (ACY-loaded CP—white circle). The data of weight loss (%) are expressed as a function of the time, and as the mean value of three different experiments ± standard deviation. *p < 0.05.

Observing Fig. 5, it is possible to note that after 8 h from the start of the experiment, the percentages of weight loss from the empty cubic phase and the Acyclovir-loaded cubic phase were not significantly different, recording respectively 33.30 ± 3.11% and 27.92 ± 1.43% of weight loss. The only difference that is found is rather in the degradation kinetics which is slightly faster in empty sample than in Acyclovir-loaded cubic phase. Obviously, these data can be considered only preliminarily. We are aware that the environment to which the formulation would be exposed in vivo will be very different from that simulated in the experiment: the presence of physiological enzymes of the eye and pathological enzymes of the viral agent, could in fact accelerate the degradation process.

**Figure 6:**
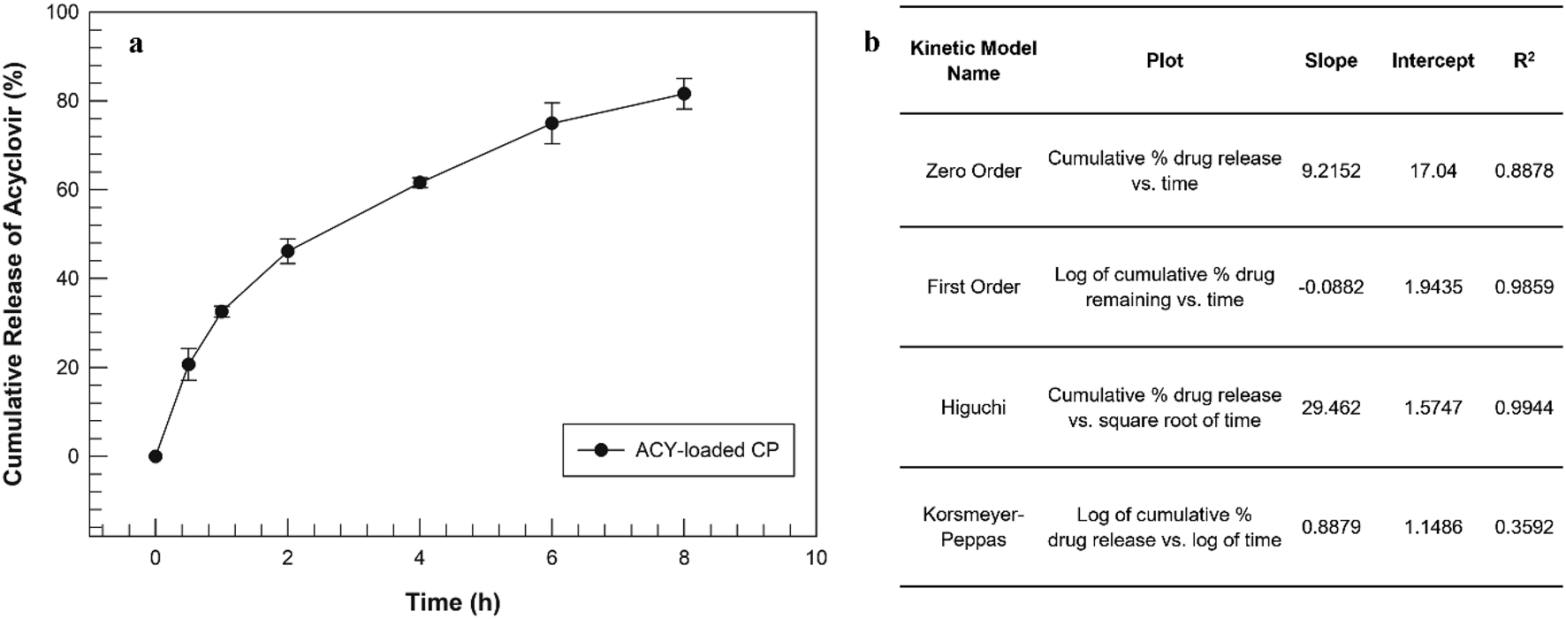
Release of Acyclovir from cubic phase in simulated tear fluid (**a**) and the square-root of time relationship (**b**). The data are expressed as the mean value of three different experiments ± standard deviation.

Nevertheless, the obtained degradation profile for Acyclovir-loaded cubic phase was noteworthy especially when it was analyzed as a function of the drug release profile, reported in Fig. 6.

**Figure 7:**
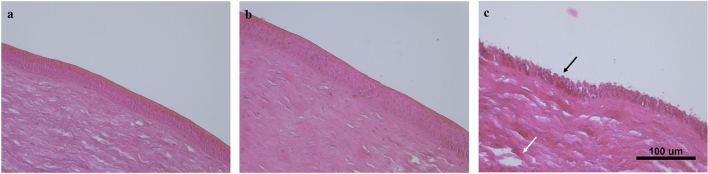
Histological evaluation of cornea sections after 24 h of simulated tearing, with hematoxylin and eosin staining (magnification 200 ×). Panel (**a**) shows intact corneal epithelial layer following the application of STF, while panel (**b**) reports cornea section treated with 100 µL of in situ forming cubic phase. Panel (**c**) is representative of chemically induced damage on porcine cornea following the application of NaOH 0.1 N for 10 min. Black and white arrows indicate damaged epithelium and disorganized stroma respectively.

Also in this case, we used simulated tear fluid to mimic the ophthalmic environment. The release profile showed a faster release in the first hours of the experiment, reaching after 8 h a percentage of 81.60% ± 3.45 of the total amount of Acyclovir incorporated into precursor lamellar phase (Fig. 6a). The release of a drug from such a well-structured matrix can depend on different factors; of course, the physico-chemical properties of the drug and the structure of the cubic phase play an important role. In fact, depending on drug properties and on its affinity, it may be placed in the lipid structures or in the aqueous channels and this localization may influence the rate and the grade of drug release^10^. In the case of Acyclovir, its solubility permitted us to hypothesize its double disposition both in aqueous channels and in lipid compartments, thus inducing a good release profile from cubic phase during the first 8 h of experiments. To better understand the release mechanism of Acyclovir from loaded cubic phase, the collected data were plotted according to four release kinetic models (zero order, first order, Higuchi and Korsmeyer–Peppas)^31^. As shown in Fig. 7b, the best match was obtained with the Higuchi model with a linear correlation coefficient (R^2^) of 0.9944, indicating a diffusion-controlled release^32^.

In fact, if Acyclovir had only established strong interactions with GMO, its release would have been slower and incomplete, and the release profile would have mirrored that of degradation one as well as for lipophilic drugs^33^.

As already mentioned, when a cubic phase is completely formed its state of aggregation results very stable, not undergoing structural changes when environmental conditions are modified, such as addition of medium or temperature variation. This peculiarity of cubic phase could be very advantageous for ophthalmic application: the physiological hydration state of application site does not alter the properties of the in situ formed system, making the release profile of acyclovir reported in Fig. 7 predictable and very close to eventual in vivo release.

### Ex vivo investigations: histological examination

In this study we proposed lyotropic liquid crystals for ophthalmic application because we demonstrated, starting from rheological data, that lyotropic liquid crystals can transit from an easily applicable lamellar phase to a more viscous cubic phase, also in presence of Acyclovir. It is necessary to remember that main limits of ophthalmic disease treatments are the rapid precorneal clearance of drug(s) and the reduced residence time; in fact, it was estimated that only 5–10% of administered dose can enter the eye depending on the drug’s permeability coefficient and molecular weight^34,35^. In this scenario, lyotropic liquid crystals can be considered as a solution because of their ability to perfectly adhere on the ocular surface and to resist the lacrimation. To confirm this capability, we isolated porcine cornea and lyotropic liquid crystals as lamellar phase were applied on this tissue. In Supplementary Video S1, we demonstrated the really easy application of lamellar phase in its liquid form. Moreover, as shown, the applied lamellar phase converts itself to cubic phase when it makes contact with the corneal surface and without flowing out of the application site. In this ex vivo study, we also confirmed that in situ formed cubic phase can perfectly adhere at the ocular surface and to resist at gravity force, without falling off when the tissue has been flipped (Supplementary Video S2). In Supplementary Video S3, when a prolonged STF dripping was performed, simulating a lacrimal process, the in situ formed cubic phase containing Methylene Blue did not lose any trace of probe, as confirmed by the absence of signals in UV–Vis spectra.

After confirmed the real ability of lamellar phase to in situ transform in cubic phase following administration on porcine cornea and its ability to bear the solicitation without leaving the ocular surface, we carried out histopathological analysis on porcine cornea to evaluate if in situ forming cubic phase can induce a damage on tissue. The results were compared both with a negative control, represented by untreated isolated corneas, and with a positive control, consisting of chemically damaged corneas following a treatment with NaOH 0.1 N for 10 min.

The healthy and untreated cornea (Fig. 7a) exhibited a uniform structure with visible orderly arranged epithelial cells and organized stroma. Comparing untreated cornea section with treated ones (Fig. 7b), it is possible to note that no histopathological changes nor infiltration of inflammatory cells occurred when porcine cornea was treated with 50 µL of in situ forming cubic phase. In presence of tested formulation, the epithelial and stroma structures were also maintained as unaltered, unlike what happens when porcine cornea was treated with a patch soaked in NaOH 0.1 N for 10 min (Fig. 7c) confirming the great safety profile of GMO-based formulation for ophthalmic administration^36,37^. The desired and chemically induced damage led to an evident destruction of the corneal epithelium (black arrow) and a disorganization of stroma (white arrow).

These results confirmed no toxicity of the formulation proposed in this experimental work once applied to the porcine cornea.

## Conclusion

In this research work we proposed and confirmed the applicability of lyotropic liquid crystals as drug delivery systems for ophthalmic administration of acyclovir. Despite acyclovir was chosen as a model drug, it is characterized by specific physico-chemical properties which it shares with many ophthalmic active ingredients. The results showed the ability of lamellar phase to contain a suitable amount of acyclovir and to transform itself in cubic phase when it is posed in contact with excess hydration medium. This aspect is very important because the lowest viscosity of the lamellar phase could permit it to be easily administered in drops form on ophthalmic surface, on the contrary the highest viscosity and the greater stability of the in situ formed cubic phase could enable the optimization of any ophthalmic therapy. Moreover, the ex vivo studies confirmed the high adhesion force of in situ formed cubic phase on porcine cornea, even during a forced drip of STF, confirming its resistance during the simulated lacrimation. Last, but not least, ex vivo histopathological studies showed no differences in cornea structures when untreated and treated cornea were compared. These results confirmed the safety of in situ forming cubic phase application on ophthalmic tissue. Of course, other experiments are necessary and more drugs with a potential to treat ophthalmic diseases should be tested, but the already obtained results confirmed our hypothesis to propose lyotropic liquid crystals for ophthalmic application and for delivery of active molecules such as acyclovir itself.

## Materials

Glycerol monooleate (GMO, 98 wt% monoglycerides, 1.5 wt% diglycerides, and 0.4 wt% free fatty acids) was purchased by BASF Catalysts Germany GmbH (Nienburg, Germany). Acyclovir (9-[(2-Hydroxyethoxy) methyl] guanine), sodium hydrogen carbonate (NaHCO_3_), sodium chloride (NaCl) and calcium chloride (CaCl_2_) were provided by Sigma Aldrich (Milan, Italy). Cellulose membrane Spectra/Por (Cut off 50 kDa) were obtained from Spectrum Laboratories Inc., NJ, USA. Deionized double-distilled water was employed during the investigations. All other materials and solvents used in this research were of analytical and HPLC grade and did not need further purification.

## Methods

### Preparation of lamellar phases and sterilization in autoclave

The empty lamellar phases were obtained mixing GMO and distilled water in an 80:20 (% w/w) ratio. The amount of GMO was pre-heated at 45 ± 1 °C and maintained under continuous and mild stirring by using a magnetic stirrer, then the water, retained at the same temperature, was added dropwise. The same protocol was observed to obtain the loaded lamellar phase: Acyclovir (1 mg/g) was added by dissolving the right amounts in the amphiphilic constituent and in aqueous medium, according to drug’s solubility in GMO and water^38,39^, respectively.

To evaluate the possibility to sterilize samples, empty lamellar phases were subjected to heating at 121 °C for 15 min at 2 bar pressure, in an autoclave system^40^. After autoclave cycles, rheological analysis was newly carried out to evaluate any change in rheological behaviour of samples.

### In vitro tear fluid uptake

The transition from lamellar to cubic phase was in vitro induced through a gravimetric method^41^ by adding an excess (5 mL) of a simulated tear fluid (STF) to sterilized lamellar phase (1 mL) until a complete swelling to cubic phase was observed. The STF was prepared by dissolving sodium chloride (0.67% w/v), sodium hydrogen carbonate (0.2% w/v) and calcium chloride (0.008% w/v) in water and pH adjusted to 7.4^42,43^. In detail, the known amount of LLC lamellar phase was weighted in a glass vial, before adding the STF. To mimic the eye's temperature^44^, the system was maintained at 35 ± 1 °C, until the hydration equilibrium was reached. Then no absorbed STF was removed, and the vial weighted again. The increase in vials’ weights was correlated to the tear fluid uptake (TFU) by the lamellar phase in its transition to total swollen cubic phase. The TFU% was calculated, according to Eq. (1):

1 $$ TFU\% = \frac{{W_{CP} - W_{LP} }}{{W_{LP} }} \times 100 $$

where W_CP_ and W_LP_ were the weight of vial containing the swollen cubic phase obtained after adding the aqueous medium and the weight of vial containing the lamellar phase before inducing the transition, respectively.

### Phase transition study

To verify texture and anisotropy of the lyotropic liquid crystalline phases, a microscopical characterization of lamellar and cubic phases were assessed using Morphologi G3-S microscope equipped with the optical system Nikon^®^ CFI 60 Brightfield/Darkfield. In detail, each sample (10 μL lamellar phase and 10 mg of cubic phase) was loaded directly above instrument’s plate to avoid any possible mechanical stress and covered with a coverslip glass (20 × 20 mm) to ensure the maintenance of the GMO hydration. The bottom light was polarized using two crossed polarizing filters which were perpendicularly placed above the light source to form a 90° angle to each other. Structures photographs were captured using 10 × or 50 × magnifications and thus exported as TIFF images by means of Morphologi software.

### Rheological measurements

The rheological behaviour of formulations was investigated at room (25 ± 1 °C) and ophthalmic (35 ± 1 °C) temperatures using Kinexus^®^ Pro Rotational Rheometer equipped with thermo-controlled system. Frequency sweep measurements were carried out evaluating the mechanical parameters (elastic modulus G′, viscosity modulus G″ and complex viscosity η*) in a range of frequency (0.1–10 Hz) and under a constant shear stress (1 Pa). The analysis was performed by means of a cone-plate geometry (diameter 40 mm; angle 2°; gap between geometry and cartridge of 1 mm) and a plate-plate geometry (diameter 20 mm; gap between geometry and cartridge of 1.2 mm) for lamellar and for cubic phase samples, respectively. Before starting the measurements, each sample was carefully loaded on the lower cartridge and left to rest for 5 min, thus the loading procedure could not affect the results^45^.

### Turbiscan^®^ lab expert stability analysis

Stability studies over time of both empty and loaded lamellar phases were assessed by using Turbiscan^®^ Lab Expert apparatus, equipped with Turbiscan Lab Cooler. The stability measurements were carried out at 25 ± 1 °C, using a pulsing near infrared LED set at a wavelength of 880 nm for 3 h^46^, time during whom integrated TurbiSoft software recorded variations of delta-backscattering (ΔBS) and delta-transmission (ΔT) profiles as function of time and sample height^47^.

### In vitro Acyclovir release from cubic phase

Dialysis method with cellulose acetate membrane (Cut off 50 kDa) was chosen to investigate Acyclovir release profile from cubic phase. A cellulose bag was filled with 1 mL of Acyclovir-loaded lamellar phase, closing the ends with suitable clips and thus it was immersed into acceptor release medium (STF, 100 mL, pH 7.4), maintained at 35 ± 1 °C under continuous stirring. At prefixed times after the phase transition (0, 30 min, 1, 2, 4, 6, 8, 24, 48 h), 1 mL of acceptor medium was removed and aliquoted, then the same volume was replaced with fresh acceptor medium. Once all collected aliquots, they were analysed by using HPLC apparatus.

### In vitro spontaneous degradation of cubic phase

The degradation rate of both empty and loaded cubic phases was evaluated through a gravimetric investigation using STF (pH 7.4, 35 ± 1 °C) as degradation medium. Firstly, 1 g of lamellar phase was weighted in a small beaker and 5 mL of STF was added to favour a complete transition to totally swollen cubic phase. After the formation of stable cubic phase, the amount of no absorbed STF was removed and the beaker weighted (*t0*). Then, the beaker was refilled with 5 mL of fresh medium and pre-equilibrated at 35 ± 1 °C. At any pre-fixed time (*tx*), the beaker containing the dry cubic phase was weighed before adding fresh medium again. The weight loss of cubic phase over time was used as a parameter of spontaneous degradation in simulated tear fluid. The percentage of weight loss was expressed according to Eq. (2):

2 $$ Weight\;loss\;(\% ) = 100 - \left[ {\left( {\frac{{W_{tx} }}{{W_{t0} }}} \right) \times 100} \right] $$

where *W*_*tx*_ was the weight of dry cubic phase at each fixed time, while *W*_*t0*_ was weight of newly formed cubic phase before each addition of STF degradation media at pre-fixed times.

### Ex vivo adhesion evaluation

In order to better observe the in situ formation of cubic phase and its mucoadhesive properties, we carried out ex vivo tests on isolated porcine corneas. The fresh eyeballs were kindly provided by local slaughterhouse, and they were maintained in Hanks’ balanced salt solution containing penicillin/streptomycin during the transport to the lab. The cornea was isolated starting from undamaged eyes as previously described by Van den Berghe et al.^48^.

A suitable amount of lamellar phase (50 µL) was applied on isolated cornea surface and the in situ transition in cubic phase was observed. To confirm the ocular adhesion of formulation, a STF solution was dripped onto the formulation, and the loss of cubic phase was monitored.

### Evaluation of the safety of the formulation on porcine cornea

The eyeballs were mounted on a support suitably constructed in such a way that the bulb was immersed in its posterior portion in a maintenance liquid and the cornea exposed to a pumping system that wanted to simulate tearing with a drip rate equal to 0.1 mL/min^49^.

For evaluating the effects of in situ forming cubic phase, the available eyeballs were divided in three groups (n.3 for each group). The first group was used as negative control and the eyeballs were untreated and maintained under simulated tearing for the entire experiment, keeping them moist. A second group of fresh eyeballs was used as positive control of corneal damage. In detail, the cornea of these eyeballs has been deliberately and chemically damaged by applying a soaked patch of 0.1 N NaOH for 10 min^16^ and subsequently they were maintained under simulated tearing. Finally, a third group of porcine eyeballs was used to test the formulation. 100 µL of lamellar phase was applied on cornea and, following its instantaneous transition to cubic phase, the treated eyeballs have been subjected to simulated tearing for 24 h.

After 24 h, the cornea of all eyeballs was dissected as already described and fixed in 10% neutral-buffered formalin solution for 24 h and transferred into 75% ethanol until processed. Parallel slices of cornea were sectioned and embedded in paraffin. Histological sections of 6 µm thickness were cut, deparaffinized with xylene, rehydrated with aqueous solutions of decreasing ethanol concentrations, and stained with hematoxylin and eosin (H&E). They were washed properly with PBS and fixed in 10% formaldehyde in PBS.

### HPLC analysis

A HPLC apparatus equipped with a PU-4180 RHPLC quaternary pump, a UV-4070 UV–Vis, an AS-4050 autosampler and an LC-NetII/ADC interface box (Jasco Europe, Lecco, Italy) was used to assess the analytical quantification of Acyclovir, The samples were eluted under isocratic conditions using an RPC18 column (Mediterranea SEA18 column, size 250 mm × 4.6 mm, 5 µm) operating at room temperature (25 °C) under a flow of 1 mL min^−1^, and the drug was detected at 251 nm with a retention time of 11,6 min. A mixture of water and acetonitrile (97:3 v/v) containing 0.05% (v/v) of trifluoroacetic acid was filtered through a 0.2 μm polycarbonate filter and used as the mobile phase^50^. Data were acquired and processed with ChromNAV v.2.04.01 software (Jasco Inc., Tokyo, Japan), using the linear equation y = 4521.7x + 129,384 with a correlation coefficient (R^2^) of 0.9999 obtained from the analysis of standard solutions (concentration range of 0.25–25 µg/mL, n = 6). Under the experimental conditions, the limit of detection (LoD) and limit of quantitation (LoQ) were 0.08 µg/mL and 0.25 µg/mL, respectively.

The detection of Methylene Blue was carried out by using a PerkinElmer Lambda 35 ultraviolet–visible (UV–Vis) spectrophotometer at a λmax of 660 nm^51^.

### Statistical analysis

Statistical analysis of the various experiments was performed by one-way ANOVA, considering a p value of < 0.05 (*) and of < 0.001 (**) as significant. A posteriori Bonferroni t-test was carried out to check the ANOVA test.

### Supplementary Information

Supplementary Information.

Supplementary Video S1.

Supplementary Video S2.

Supplementary Video S3.

## Data Availability

All data generated or analysed during this study are included in this published article and its supplementary information files.
