# Author Correction: Perspective use of bio-adhesive liquid crystals as ophthalmic drug delivery systems

**DOI:** 10.1038/s41598-023-49738-2

**Published:** 2023-12-18

**Authors:** Martine Tarsitano, Antonia Mancuso, Maria Chiara Cristiano, Konrad Urbanek, Daniele Torella, Donatella Paolino, Massimo Fresta

**Affiliations:** 1 Department of Health Science, University Magna Graecia of Catanzaro, Campus Universitario‑Germaneto, Viale Europa, 88100 Catanzaro, Italy; 2 Department of Experimental and Clinical Medicine, University Magna Graecia of Catanzaro, Campus Universitario‑Germaneto, Viale Europa, 88100 Catanzaro, Italy; 3 Department of Medical and Surgical Sciences, University Magna Graecia of Catanzaro, Campus Universitario‑Germaneto, Viale Europa, 88100 Catanzaro, Italy; 4 Department of Molecular Medicine and Medical Biotechnologies, University of Naples “Federico II”, Via A. Panzini 5, 80131 Naples, Italy; 5 CEINGE-Advanced Biotechnologies, Via G. Salvatore 486, 80131 Naples, Italy

Correction to: *Scientific Reports* 10.1038/s41598-023-42185-z, published online 27 September 2023

The original version of this Article contained errors in the names of the authors Martine Tarsitano, Antonia Mancuso, Konrad Urbanek, Daniele Torella, Donatella Paolino & Massimo Fresta, which were incorrectly given as Tarsitano Martine, Mancuso Antonia, Urbanek Konrad, Torella Daniele, Paolino Donatella & Fresta Massimo.

The original Article has been corrected.

