# Supplementary material for: Perspective use of bio-adhesive liquid crystals as ophthalmic drug delivery systems
Source: Sci Rep. 2023 Sep 27;13:16188. doi: 10.1038/s41598-023-42185-z (PMC10533901; doi:10.1038/s41598-023-42185-z)
Supplement: Supplementary file 1 — Supplementary Information. [file 41598_2023_42185_MOESM1_ESM.pdf]

Supplementary Table S1

	Complex viscosity (η - kPa·s) of empty lamellar phase	
Frequency values (Hz)	Before autoclaving process	After autoclaving process
0.1	6.32 ± 0.23	3.55 ± 0.15 (**)
1	5.45 ± 0.12	3.21 ± 0.27 (**)
10	2.07 ± 0.023	3.71 ± 0.90 (*)

Supplementary Table S1. Complex viscosity values obtained at specific frequency of LLC lamellar phase analyzed at 25 ± 1°C before and after sterilization process, carried out by using an autoclave (120°C for 15 min). The results are expressed as mean value ± standard deviation. *$p<0.05$ and **$p<0.001$ for complex viscosity of lamellar phase before autoclaving process vs complex viscosity values after sterilization.

Supplementary Figure S1

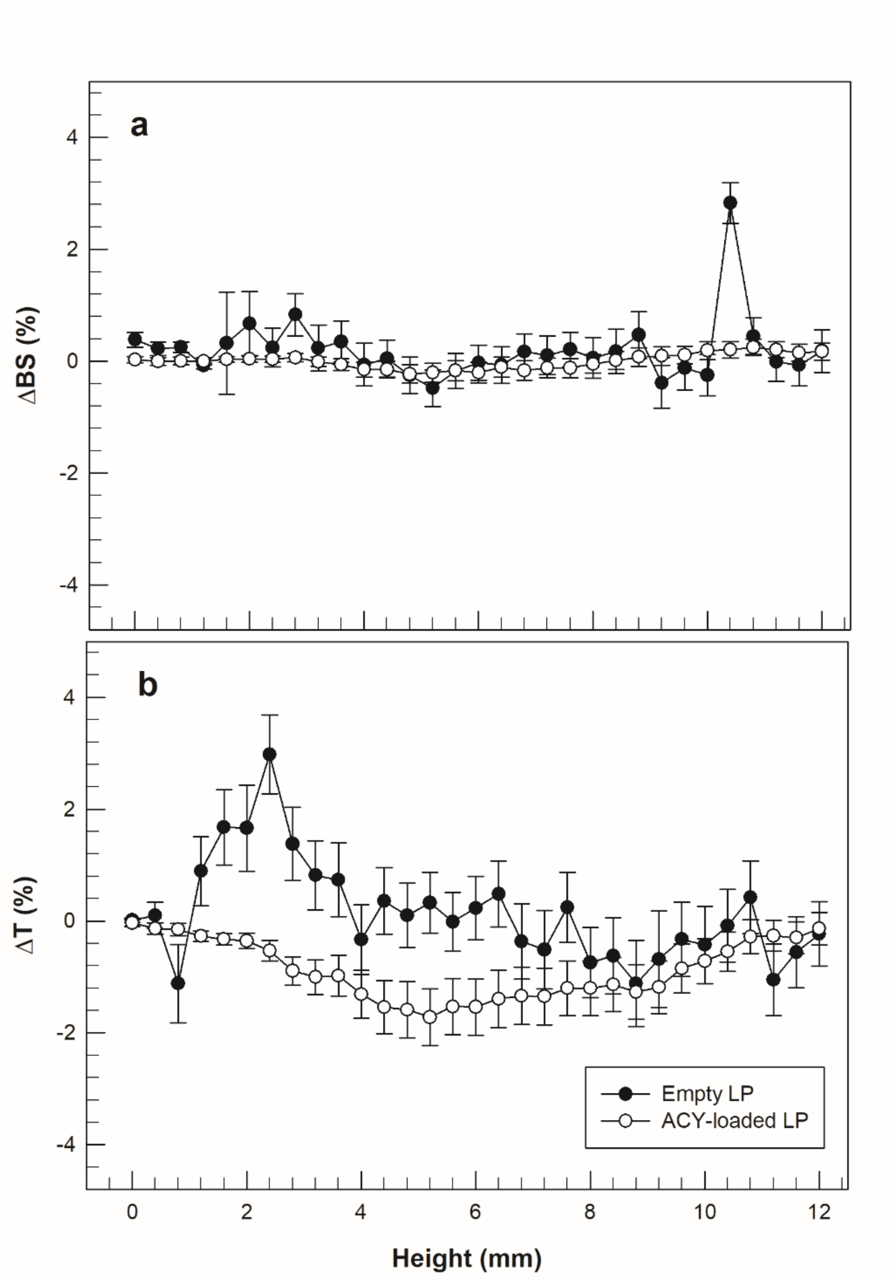

Supplementary Figure S1. Delta back scattering (ΔBS —a) and delta transmission (ΔT —b) of empty and ACY-loaded lamellar phases. Panels report representative data from five independent experiments. Data are reported as a function of time (0–3 h) and sample height.
